# Impact of positive end-expiratory pressure on autonomic nervous system activity and its interaction with cerebrovascular reactivity – an experimental study

**DOI:** 10.1007/s10877-026-01420-4

**Published:** 2026-02-18

**Authors:** Agnieszka Uryga, Magdalena Kasprowicz, Marek Czosnyka, Agnieszka Kazimierska, Rønnaug Hammervold, Shirin K. Frisvold

**Affiliations:** 1 Department of Biomedical Engineering, Faculty of Fundamental Problems of Technology, Wroclaw University of Science and Technology, Wroclaw, Poland; 2 Division of Neurosurgery, Department of Clinical Neurosciences, University of Cambridge, Cambridge, UK; 3 Institute of Electronic Systems, Warsaw University of Technology, Warsaw, Poland; 4 Department of Anesthesia and Intensive Care, Nordland Hospital Trust, Bodø, Norway; 5 Department of Clinical Medicine, Faculty of Health Sciences, UiT The Arctic University of Norway, Tromsø, Norway; 6 Research Laboratory, Nordland Hospital Trust, Bodø, Norway; 7 Department of Anesthesia and Intensive Care, University Hospital North Norway, Tromso, Norway

**Keywords:** PEEP, Autonomic nervous system, Baroreceptors, Cerebrovascular reactivity

## Abstract

Mechanical ventilation requires positive end-expiratory pressure (PEEP), which has shown to improve lung recruitment and gas exchange. However, the increase in intrathoracic pressure might impact cardiac function and autonomic nervous system (ANS) activity. This post hoc analysis aimed to evaluate the impact of incremental PEEP on ANS activity and to examine its relationship with cerebrovascular reactivity in an experimental model. Twelve anesthetized, mechanically ventilated pigs were studied in both the supine and prone positions at baseline PEEP (5 cmH₂O) and at incremental PEEP levels (10, 15, 20 cmH₂O). ANS parameters included time-domain heart rate variability (HRV): the standard deviation of normal pulse intervals (SDNN), the root mean square of successive differences between normal heartbeats (RMSSD), HRV entropy measures: fuzzy entropy (FuzzyEn) and sample entropy (SampEn), heart rate asymmetry indices: Guzik’s index (GI) and Porta’s index (PI), and baroreflex sensitivity (BRS). Cerebrovascular reactivity was assessed using the pressure reactivity index (PRx). Relationships between ANS metrics and PRx were analyzed using univariate and multivariate linear mixed-effects models (LMEMs), adjusted for PEEP. Increasing PEEP significantly altered HRV entropy (FuzzyEn: p = 0.006; SampEn: p = 0.018) and BRS (p = 0.013) in the supine position, with similar trends observed in the prone position. In the univariate LMEM analyses, all ANS metrics significantly influenced PRx in both positions. However, in the multivariate LMEM models, only SDNN (p = 0.001), RMSSD (p = 0.001), GI (p = 0.003), and PI (p = 0.007) remained significantly associated with PRx in the prone position, whereas no ANS metrics remained significant in the supine position. Incremental PEEP modifies ANS activity, as reflected by changes in HRV entropy and BRS. Prone positioning appears to preserve the autonomic modulation of cerebrovascular reactivity under PEEP. These findings underscore the importance of considering ventilatory settings when assessing heart–brain interactions.

**Supplementary Information:**

The online version contains supplementary material available at 10.1007/s10877-026-01420-4.

## Introduction

Mechanical ventilation in intensive care unit (ICU) patients commonly involves the application of positive end-expiratory pressure (PEEP) to enhance lung recruitment and improve gas exchange. PEEP alters airway, pleural, and transpulmonary pressures, leading to the redistribution of blood volume within the puCrick SJ,lmonary circulation. The associated increase in intrathoracic pressure affects venous return, cardiac preload and afterload, as well as arterial blood pressure, all of which are closely linked to autonomic nervous system (ANS) regulation and cerebral hemodynamics.

The ANS plays a central role in maintaining cardiovascular homeostasis [34]. Its activity is commonly assessed using indices such as baroreflex sensitivity (BRS) and heart rate variability( HRV) [57, 68]. Significant alterations in HRV or reductions in BRS have been associated with increased mortality, morbidity, and intracranial hypertension in critically ill patients [53, 61, 72–74]. The modulation of ANS activity by PEEP-assisted breathing or mechanical ventilation remains a matter of debate and has not been extensively studied [13, 78, 79]. In healthy subjects, the application of PEEP at 5 cmH_2_O has been shown to increase the high-frequency index of the spectral baroreflex gain compared with control conditions without PEEP [26, 27]. Conversely, a randomized controlled trial demonstrated that higher nasal positive airway pressure was associated with a reduction in the mean slope of spontaneous baroreceptor sequences [77]. However, despite the crucial role and frequent dysfunction of ANS in critically ill patients, the assessment of ANS activity is not a standard procedure in the ICU, due to its complexity, variability, and the lack of easy, universally applicable bedside tools [1, 32]. Moreover, the interaction between ANS activity and respiratory mechanics remains unclear. Very few studies have examined cardiac autonomic activity in the supine and prone positions, because the associated changes in cardiopulmonary mechanics and baroreflex function can shift autonomic balance and influence monitoring and clinical risk assessment.

Autoregulation of cerebral blood flow refers to the brain’s ability to maintain stable cerebral blood flow despite fluctuations in cerebral perfusion pressure [9, 39]. In patients with traumatic brain injury, this autoregulatory mechanism may be impaired, leading to hypoperfusion or hyperemia [20]. Cerebrovascular pressure reactivity refers to the ability of vascular smooth muscle cells to respond to changes in transmural pressure [91] and is considered a primary mechanism underlying the autoregulation of cerebral blood flow [11]. The pressure reactivity index (PRx), derived from arterial blood pressure and intracranial pressure using time correlation of their spontaneous fluctuations, is a widely used measure of cerebrovascular pressure reactivity. In clinical practice, it is used to determine continuous optimal cerebral perfusion pressure [2–4]. Current evidence suggests that under controlled conditions, with stable arterial blood pressure and strict CO_2_ control, increasing PEEP does not have a clinically relevant effect on cerebral autoregulation, whether assessed by PRx [2, 3, 28, 30] or the mean velocity index (Mxa) [66].

Recent studies have highlighted the complex cross-talk between the brain, heart, and lungs [47, 62]. Increased intrathoracic pressures, induced by PEEP changes, may contribute to this interaction through hemodynamic, inflammatory, and neurohormonal effects [31, 33]. Experimental and clinical evidence suggest that cerebrovascular reactivity and ANS activity are interrelated [10, 52, 83, 88]. This interaction may differ between healthy individuals and patients with acute brain injury or systemic hemodynamic disturbances [40, 49, 75, 76]. Following traumatic brain injury, autonomic failure and cerebrovascular reactivity impairment are both associated with poor outcomes [12, 40]. Previous studies have indicated that autonomic responses can modulate cerebrovascular reactivity, especially under conditions of pathological intracranial changes, such as increased intracranial pressure [25, 74–76]. Nevertheless, the influence of ANS activity on cerebrovascular reactivity under conditions of incremental PEEP changes has not been investigated.

Therefore, we hypothesized that increasing intrathoracic pressure alters autonomic regulation and modifies the coupling between ANS activity and cerebrovascular reactivity. The primary aim of this study was to investigate the effects of incremental increases in intrathoracic pressure, induced by PEEP, on ANS activity in the prone and supine positions. The secondary aim was to determine whether and how PEEP changes modulate the relationship between cerebrovascular pressure reactivity and autonomic cardiovascular control under controlled experimental conditions.

## Materials and methods

### Ethics statement

The study was conducted in strict compliance with the Norwegian Laboratory Animal Regulations and the EU Directive 2010/64/EU to ensure the humane treatment of animals. Ethical approval was obtained from the Norwegian Animal Research Authority (FOTS ID 27107). The ARRIVE guidelines checklist for the reporting of animal research was used.

### Study design

This study is a retrospective secondary analysis of data from previously conducted randomized crossover experiments. Although these data were part of our earlier publication [30], the current study focused on the effect of PEEP changes on ANS activity, an aspect not previously analyzed. The animal protocol has been described in detail previously [30]. Twelve anesthetized Norwegian domestic landrace pigs (11 males, 1 female), each weighing approximately 25 kg, were included. The pigs were randomized to start in either the supine or prone position, with the order reversed during the experiment. PEEP levels were adjusted every 20 min across 5, 10, 15, and 20 cmH_2_O. Each pig was analyzed separately in both the prone and supine positions across each of the PEEP levels.

### Animal experiments and data monitoring

Animals were sedated immediately after removal from the pen using an intramuscular injection of ketamine (20 mg/kg), midazolam (0.5 mg/kg), and atropine (1 mg). Anesthesia was then induced with an intravenous infusion of morphine (2 mg/kg/h), midazolam (0.15 mg/kg/h), and pentobarbital (4 mg/kg/h). Animals were intubated using a Portex endotracheal tube (ID 6.5 to 7.0-mm; Smiths Medical International Ltd., Kent, United Kingdom). Depth of anesthesia was regularly assessed according to standard protocols using the pedal withdrawal reflex, palpebral reflex, and jaw tone. Anesthetic agents, dosing, and sedation depth were kept constant throughout baseline and intervention periods (i.e., across PEEP levels).

Mechanical ventilation was initiated using a Datex-Ohmeda Engström Carestation intensive care ventilator (GE Healthcare, Madison, WI) in volume-control mode, with a tidal volume of 9 mL/kg, fraction of inspired oxygen (FiO_2_) of 0.40, PEEP of 5 cmH_2_O, an inspiratory-to-expiratory ratio of 1:2, and an inspiratory pause 20%. The respiratory rate (20–30 breaths/min) was adjusted to maintain normocapnia (PaCO₂ 35–45 mmHg), particularly when tidal volume was reduced at higher PEEP levels to limit airway pressures. A FluxMed esophageal catheter (MBMED, Martinez, Argentina) was placed and connected to the ventilator to monitor esophageal pressure (P_es_).

To ensure euvolemia, an intravenous fluid bolus of 500 mL of Ringer’s acetate (Fresenius Kabi, Oberdorf, Switzerland) was administered at a rate of 30 mL/kg/h, followed by a 5 mL/kg/h continuous infusion throughout the study.

The MP70 monitors continuously acquired data on mean arterial blood pressure (MAP), central venous pressure (CVP), and pulmonary artery blood pressures (PAP), systolic blood pressure (SBP), diastolic blood pressure (DBP), electrocardiography (ECG), heart rate (HR), respiratory rate, end-tidal carbon dioxide (EtCO_2_), saturation, and body temperature. Cardiac output (CO) was monitored using a thermodilution system (Edwards Lifesciences Corporation). Extravascular lung water and stroke volume variation (SVV) were calculated by the pulse contour cardiac output (PiCCO) systems (Pulsion Medical Systems, Getinge, Feldkirchen, Germany). The monitoring also included esophageal pressure with a balloon catheter (FluxMed, MBMED, Argentina) connected to the ventilator (Engström Carestation, GE Healthcare). ICP was measured using a Codman MicroSensor ICP transducer inserted into the left frontal hemisphere and connected to a Codman ICP Express monitor (Codman & Shurtleff, MA, USA).

### Data processing

Intensive Care Monitoring software (ICM + , Cambridge Enterprise, Cambridge, United Kingdom) was used for high-resolution signal acquisition at a frequency of 500 Hz. All the data were preprocessed in ICM + . The recordings were visually inspected and cleaned for artifacts. The signals were downsampled by nonoverlapping 10-s averages.

### Respiratory mechanics variables

Data from the ventilator was extracted from ICM + software as one value per monitoring period. The following variables were retrieved as absolute values: end-inspiratory (Paw_ei_) and end-expiratory (Paw_ee_) airway pressure, end-inspiratory (Pes_ei_) and end-expiratory (Pes_ee_) esophageal pressure, peak pressure of the respiratory system (Ppeak_rs_) and chest wall (Ppeak_cw_) being the maximal values of Paw and Pes, respectively. The following variables were calculated: peak pressure across the lung (Ppeak_l_) = Ppeak_rs_ − Ppeak_cw_, end-inspiratory transpulmonary pressure (TPP_ie_) as the difference between Paw_ei_ and Pes_ei_, and end-expiratory transpulmonary pressure (TPP_ee_) by subtracting Pes_ee_ from total end-expiratory airway pressure.

### Cerebrovascular reactivity index and autonomic nervous system metrics

**Fig. 1:**
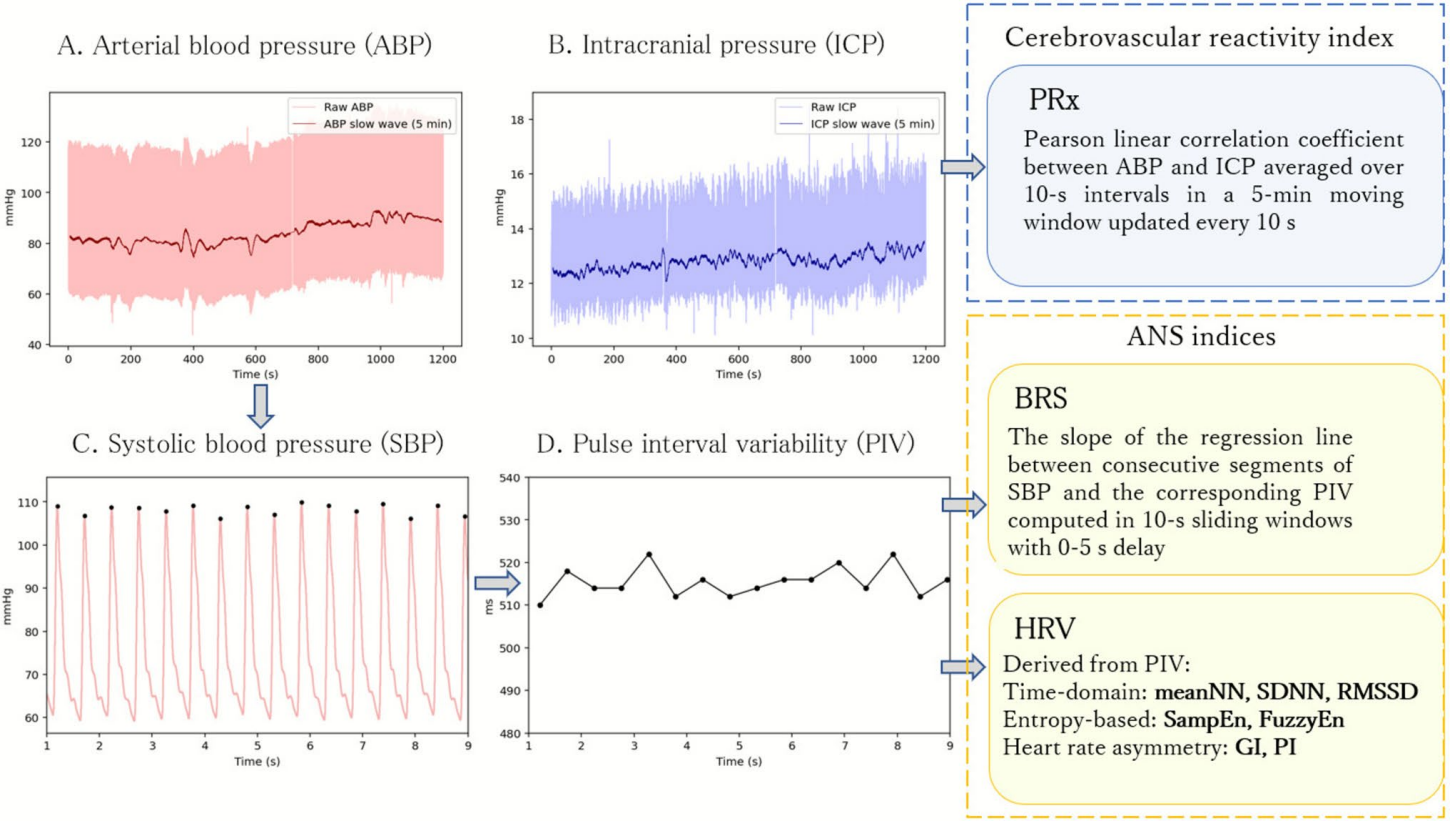
Graphical summary of physiological signal processing and index derivation. Raw arterial blood pressure (ABP) and intracranial pressure (ICP) waveforms are used to compute cerebrovascular reactivity (PRx) from slow-wave oscillations. Systolic blood pressure (SBP) and pulse interval variability (PIV), both extracted from the ABP signal, are used to estimate baroreflex sensitivity (BRS), while heart rate variability (HRV) metrics are derived exclusively from PIV. The presented raw signals were recorded during a 20-min period at a positive end-expiratory pressure (PEEP) level of 15 cmH₂O in the prone position

A concise summary of each estimated index is presented in Table 1. A graphical overview illustrating how each metric is derived from the underlying physiological signals is shown in Fig. 1. Below, we provide the relevant definitions of all estimated metrics.

**Table 1:** Description of the autonomic nervous system and cerebrovascular reactivity indices applied in the study

Metric	Acronym	Unit	Description	Key references
**I Cerebrovascular reactivity**				
A.** Pressure reactivity index** *Estimated from arterial blood pressure (ABP) and intracranial pressure (ICP) signals*				
Pressure reactivity index	PRx	a.u	PRx is derived from the moving correlation between slow waves of ICP (a surrogate of changes in cerebral blood volume) and mean arterial pressure (a surrogate of changes in the driving pressure). Positive PRx values indicate impaired cerebrovascular autoregulation and reflect a passive transmission of driving pressure to cerebral blood volume. Negative values are interpreted as preserved autoregulation	[21, 23, 86]
**II Autonomic nervous system**				
A.** Baroreflex sensitivity** *Estimated from pulse interval variability and systolic arterial pressure variability derived from ABP signal*				
Baroreflex sensitivity	BRS	ms/mmHg	BRS quantifies the effectiveness of the arterial baroreflex, a short-term neural feedback mechanism that maintains arterial blood pressure stability by adjusting heart rate in response to changes in blood pressure. BRS reflects the relationship between spontaneous fluctuations in systolic arterial pressure and the corresponding changes in pulse interval	[37, 38, 57]
B. **Heart rate variability metrics** All metrics below were estimated from pulse interval variability derived from ABP signal*				
**Time-domain metrics** *Quantify the amount of variability in interbeat interval*				
Mean time durations between consecutive normal heartbeats (NN intervals)	meanNN	ms	It reflects the combined influence of all regulatory mechanisms on the heart rhythm	[44, 67]
Standard deviation of NN intervals	SDNN	ms	It quantifies the combined influence of the sympathetic and parasympathetic nervous systems. In short-term resting recordings, SDNN is influenced by parasympathetically mediated respiratory sinus arrhythmia	[45, 68]
Root mean square of successive NN intervals differences	RMSSD	ms	Reflects beat-to-beat heart rate variability and is the primary time-domain measure of vagally mediated cardiac modulation	[44, 45]
**Entropy metrics** *Quantify the regularity and complexity of the interbeat interval dynamics*				
Sample entropy	SampEn	a.u	Measures the complexity of pulse interval variability by quantifying the conditional probability that similar sequences of length *m* remain similar at length *m* + 1, excluding self-matches. Lower entropy values indicate more regular and predictable interbeat intervals, often signaling a less resilient system or increased physiological stress	[16, 69]
Fuzzy entropy	FuzzyEn	a.u	Similar to SampEn, but uses fuzzy membership functions to define similarity, improving robustness to noise	[16, 17]
**Heart rate asymmetry metrics** *Describe a phenomenon of disparity in per beat acceleration and deceleration of heart rate*				
Porta’s index	PI	%	Quantifies asymmetry in the number of heart rate accelerations versus decelerations	[58, 63]
Guzik’s index	GI	%	Quantifies asymmetry in the amount of per beat change in instantaneous heart rate	[29, 63]

^*^By definition, heart rate variability is ideally derived from electrocardiography (ECG). However, due to insufficient ECG signal quality in a subset of recordings, whereas ABP signals were consistently available with adequate quality, pulse interval variability derived from ABP was used for HRV estimation

#### Cerebrovascular reactivity: pressure reactivity index (PRx)

Cerebrovascular reactivity was assessed using the pressure reactivity index (PRx), estimated from slow-wave oscillations of ABP and ICP. Mean PRx was calculated as the Pearson linear correlation coefficient between these signals (averaged over 10-s intervals) in a 5-min moving window updated every 10 s [22], see Fig. 1. Positive PRx values indicate impaired cerebrovascular autoregulation and reflect a passive transmission of arterial pressure fluctuations to cerebral blood volume, whereas negative values are interpreted as preserved autoregulation [86] (Table 1).

#### Autonomic nervous system metrics: baroreflex sensitivity (BRS)

Baroreflex sensitivity (BRS) was estimated from the ABP signal according to the method proposed by Westerhof et al. [81, 82]. BRS was calculated as the slope of the regression line between segments of systolic blood pressure and the corresponding interbeat intervals (Table 1). Because physiological variability in systolic pressure and interbeat intervals may introduce time delays between the two signals, the algorithm accounts for possible time shifts between series. BRS values were accepted when the p-value of the correlation coefficient was < 0.05, and no ectopic beats were detected [81]. All BRS calculations were performed using an algorithm implemented in the ICM + software (Fig. 1).

#### Autonomic nervous system metrics: heart rate variability (HRV)

Heart rate variability (HRV) metrics were calculated from pulse interval variability derived from ABP signal (Fig. 1). The use of ABP-derived pulse intervals was based on signal availability: ECG recordings were of insufficient quality in four of the twelve cases, whereas ABP signals of adequate quality were available in all subjects throughout the entire measurement period.

Time-domain HRV parameters included the mean duration of consecutive normal-to-normal heartbeats (meanNN; ms), the standard deviation of normal-to-normal heartbeats (SDNN; ms), and the root mean square of successive normal-to-normal heartbeats differences (RMSSD; ms). MeanNN reflects the influence of all regulatory mechanisms on heart rhythm. SDNN reflects the combined influence of both the sympathetic and parasympathetic nervous systems, and in short-term recordings is mostly modulated by parasympathetically mediated respiratory sinus arrhythmia [67]. RMSSD quantifies beat-to-beat heart rate variability and is considered the primary time-domain measure of vagally mediated cardiac modulation [5] (Table 1). MeanNN, SDNN, and RMSSD were estimated using algorithms implemented in ICM + software.

HRV entropy-based metrics were used to quantify the regularity and complexity of interbeat interval dynamics and have been shown to reflect changes in cardiac sympathetic and vagal tone. Lower entropy values indicate more regular and predictable interbeat intervals, often signaling a less resilient system or increased physiological stress [16] (Table 1). In this study, fuzzy entropy (FuzzyEn) [17] and sample entropy (SampEn) [59] (Table 1) were estimated using algorithms embedded in the Neurokit2 library [56].

Heart rate asymmetry describes the unequal contribution of heart rate accelerations and decelerations to overall HRV (Table 1). Although the underlying physiological mechanism is not fully understood, heart rate asymmetry has been linked to the balance between sympathetic and parasympathetic activity and may be influenced by breathing pattern [35, 55]. In this study, heart rate asymmetry was quantified using Porta’s index (PI) and Guzik’s index (GI) using algorithms embedded in the Neurokit2 library [56]. PI quantifies asymmetry in the number of heart rate accelerations versus decelerations [58]. GI quantifies asymmetry in the amount of per beat change in instantaneous heart rate [29].

### Statistical analysis

The distribution of the data was assessed using the Kolmogorov–Smirnov test with Lilliefors correction. Nonparametric tests were applied because the assumption of normality was not met for some of the analyzed parameters. Changes in the analyzed variables during the increase in PEEP (10, 15, and 20 cmH₂O) are expressed as differences (Δ) relative to the baseline level (5 cmH₂O). To determine whether baseline parameter values, as well as the relative differences (Δ) at each PEEP level (10, 15, and 20 cmH₂O), differed between the prone and supine positions, the Wilcoxon signed-rank test was used. The effects of the three PEEP levels on the analyzed parameters were assessed using the nonparametric Friedman ANOVA, followed by Wilcoxon signed-rank post hoc tests with Bonferroni correction, which were performed separately for each body position. Three post hoc comparisons were conducted (10 cmH_2_O vs. 15 cmH_2_O; 10 cmH_2_O vs. 20 cmH_2_O; 15 cmH_2_O vs. 20 cmH_2_O); therefore, the statistical significance threshold was set as α/3 = 0.017.

To assess the relationship between ANS metrics and PRx, while accounting for repeated measurements across all PEEP levels, we performed a series of linear mixed-effects models (LMEM) analyses [65]. First, separate univariate LMEMs were fitted for each ANS metric, with the metric as the fixed-effect predictor and PRx as the response, including subjects as a random effect. This allowed us to evaluate the independent association of each ANS metric with cerebrovascular reactivity. Next, multivariate LMEMs were constructed for each ANS metric, including both the ANS metric and PEEP level as fixed-effect predictors, and subjects as a random effect. Before fitting multivariate linear mixed-effects models, multicollinearity among explanatory variables was assessed. This approach enabled us to examine whether the associations between each ANS metric and PRx were independent of PEEP level. For both types of LMEMs models, results are reported using F statistics and corresponding p-values, which assess the statistical significance of each predictor.

Continuous variables are presented as medians with interquartile ranges (upper quartile–lower quartile). A p-value < 0.05 was considered statistically significant unless otherwise indicated. Statistical analyses were performed using STATISTICA 13 (Tibco, Palo Alto, USA) and R Statistical Software (v.4.0.2; R Foundation for Statistical Computing, Vienna, Austria).

## Results

### Neuromonitoring and ANS parameters at baseline

The baseline physiological and ANS parameter values in the prone and supine positions (*n* = 12) are presented in Table 2. HR was lower in the supine position (p = 0.015, Table 2). With respect to the ANS parameters, the meanNN was longer in the supine position, reflecting a slower HR (p = 0.023). The GI index of heart rate asymmetry was slightly greater in the pigs in the supine position (p = 0.049), and SampEn was also greater in the supine position (p = 0.012, Table 2). No significant differences were observed in the remaining parameters.

**Table 2:** Comparison of variables at a positive end-expiratory pressure (PEEP) of 5 cmH₂O (baseline) between the prone and supine positions within the same pigs. Differences were tested using the Wilcoxon test for paired (repeated) measurements. Data are presented as the median (upper–lower quartile)

Parameter	Prone position *n* = *12*	Supine position *n* = *12*	Wilcoxon p-value
*Neuromonitoring parameters*			
ICP [mm Hg]	6.7 (5.7–11.1)	11.2 (8.2–13.1)	0.433
PRx [a.u.]	-0.37 (-0.43– -0.21)	-0.09 (-0.44– -0.01)	0.480
*Respiratory parameters*			
EtCO_2_ [kPa]	5.7 (5.5–6.0)	5.7 (5.2–5.9)	0.530
Respiratory rate [breaths/min]	32 (28–34)	30 (27–32)	0.272
Paw_ei_ [cmH₂O]	12.8 (11.6–13.7)	12.5 (12.0–13.6)	0.239
Paw_ee_ [cmH₂O]	4.4 (4.2–4.6)	4.6 (4.4–4.8)	0.075
Pes_ei_ [cmH₂O]	6.9 (3.4–8.0)	8.0 (7.1–8.8)	0.136
Pes_ee_ [cmH₂O]	3.4 (1.8–4.6)	5.4 (4.8–6.1)	**0.015**
TPP_ei_ [cmH₂O]	5.9 (4.4–9.9)	4.6 (4.2–4.8)	**0.010**
TPP_ee_ [cmH₂O]	1.2 (-0.4–2.5)	-0.8 (-1.3–0.1)	**0.041**
Ppeak_rs_ [cmH₂O]	18.4 (17.1–19.9)	17.4 (16.8–20.2)	0.239
Ppeak_cw_ [cmH₂O]	8.5 (5.4–10.6)	9.6 (9.1–10.7)	0.239
Ppeak_l_ [cmH₂O]	12.5 (7.5–14.9)	7.4 (6.7–11.8)	**0.034**
*Systemic hemodynamics parameters*			
HR [bpm]	89.9 (85.1–104.8)	86.1 (75.5–99.8)	**0.015**
MAP [mmHg]	81.4 (76.8–86.3)	80.7 (78.9–84.9)	0.694
SBP [mmHg]	114.3 (109.5–118.9)	115.0 (111.7–116.4)	0.937
DBP [mmHg]	62.1 (58.3–66.1)	61.0 (58.3–66.3)	0.184
SVV [%]	5.4 (4.3–6.9)	4.6 (3.8–6.9)	0.638
CO [L/min]	3.4 (2.8–4.2)	3.4 (3.0–4.0)	0.347
CVP [mmHg]	11.5 (10.1–12.0)	13.6 (12.8–14.6)	**0.002**
PAP [mmHg]	21.5 (17.6–23.0)	21.9 (17.6–25.5)	0.307
*Autonomic nervous system metrics*			
meanNN [ms]	669 (579–713)	697.0 (602–798)	**0.023**
SDNN [ms]	8.4 (6.6–9.5)	8.3 (6.0–20.4)	0.583
RMSSD [ms]	8.4 (6.6–9.5)	8.0 (5.8–19.6)	0.583
GI [%]	50.0 (49.9–50.1)	50.1 (50.0–50.3)	**0.049**
PI [%]	50.5 (47.7–51.8)	49.9 (48.3–52.2)	0.638
FuzzyEn [a.u.]	0.38 (0.28–0.85)	0.69 (0.41–0.91)	0.182
SampEn [a.u.]	0.44 (0.28–0.77)	0.88 (0.60–1.24)	**0.012**
BRS [ms/mmHg]	5.5 (4.4–6.4)	5.2 (4.4–10.7)	0.937

ICP, intracranial pressure; PRx, pressure reactivity index; EtCO_2_, end tidal carbon dioxide; Paw_ei_, end-inspiratory airway pressure; Paw_ee_, end-expiratory airway pressure; Pes_ei_, end-inspiratory esophageal pressure; Pes_ee_, end-expiratory esophageal pressure; TPP_ei_, end-inspiratory transpulmonary pressure; TPP_ee_, end-expiratory transpulmonary pressure; Ppeak_cw_, peak inspiratory pressure of the chest wall; Ppeak_l_, lung peak pressure; Ppeak_rs_, peak inspiratory pressure of the respiratory system; HR, heart rate; MAP, mean arterial pressure; SBP, systolic blood pressure; DBP, diastolic blood pressure; SVV, stroke volume variation; CO, cardiac output; CVP, central venous pressure; PAP, pulmonary artery pressure; meanNN, mean intervals between normal R peaks; SDNN, standard deviation of the normal pulse heartbeats; RMSSD, root mean square of successive differences between normal heartbeats; GI, Guzik’s Index; PI, Porta’s Index; FuzzyEn, fuzzy entropy; SampEn, sample entropy; BRS, baroreflex sensitivity; p-values were determined using Wilcoxon test with significant results marked in bold

### Effects of PEEP modulation on neuromonitoring parameters

Changes in neuromonitoring parameters across incremental PEEP levels are presented for the prone (Table 3) and supine (Table 4) positions in all pigs (n = 12). There were no significant changes in PRx in the prone position across increasing PEEP level (p = 0.264). In the supine position, PRx decreased with increasing PEEP (p = 0.046); however no significant post-hoc differences were observed between individual PEEP levels. A detailed analysis is provided in the Supplementary Data.

**Table 3:** Changes in parameters at incremental positive end-expiratory pressure (PEEP) levels (10, 15, and 20 cmH₂O), expressed as differences (Δ) from baseline (5 cmH₂O) in the prone position (n = 12). Data are presented as medians (upper–lower quartile)

Prone position				
Relative changes of parameter	10 [cmH₂O] n = *12*	15 [cmH₂O] n = *12*	20 [cmH₂O] n = *12*	ANOVA p-value
*Neuromonitoring parameters*				
Δ ICP [mm Hg]	0.5 (0.2–1.5)	1.4 (0.6–2.5)	2.3 (1.4–4.1)^*#^	**0.001**
Δ PRx [a.u.]	0.15 (0.11–0.20)	0.18 (0.13–0.32)	0.19 (0.0–0.30)	0.264
*Respiratory parameters*				
Δ EtCO_2_ [mm Hg]	0.1 (-0.1–0.1)	0.1 (0.0–0.5)	0.4 (0.1–0.6)	**0.013**
Δ respiratory rate [breaths/min]	0.4 (-0.2–2.0)	2.2 (-0.2–4.6)	9.9 (2.3–14.6)^*#^	**0.004**
Δ Paw_ei_ [cmH₂O]	5.3 (4.7–6.1)	11.7 (10.9–12.9)^*^	19.9 (18.2–22.0)^*^	** < 0.001**
Δ Paw_ee_ [cmH₂O]	5.5 (5.3–5.7)	10.8 (10.5–11.0)^*^	16.0 (15.7–16.2)^*^	** < 0.001**
Δ Pes_ei_ [cmH₂O]	1.0 (-0.5–1.7)	4.2 (2.7–5.0)^*^	5.2 (3.3–7.7)^*^	** < 0.001**
Δ Pes_ee_ [cmH₂O]	1.6 (0.7–2.8)	4.5 (2.7–6.2)^*^	5.6 (3.4–8.1)^*^	** < 0.001**
Δ TPP_ei_ [cmH₂O]	4.4 (3.0–5.2)	8.4 (6.2–9.8)	13.7 (10.6–17.6)	**0.001**
Δ TPP_ee_ [cmH₂O]	3.4 (2.4–4.4)	6.2 (4.5–8.1)^*^	9.7 (7.2–11.8)	**0.001**
Δ Ppeak_rs_ [cmH₂O]	5.4 (4.5–6.2)	11.1 (10.6–14.7)^*^	20.9 (18.7–24.1)	** < 0.001**
Δ Ppeak_cw_ [cmH₂O]	1.3 (0.6–1.8)	4.1 (2.9–5.4)^*^	5.9 (4.0–8.2)^*^	** < 0.001**
Δ Ppeak_l_ [cmH₂O]	4.0 (3.7–5.1)	7.8 (5.8–11.6)	14.5 (10.6–18.8)	** < 0.001**
*Systemic hemodynamics parameters*				
Δ HR [bpm]	4.0 (0.3–6.2)	10.4 (3.6–17.6)^*^	25.4 (20.0–45.7)^*#^	** < 0.001**
Δ MAP [mm Hg]	-0.2 (-4.5–3.0)	0.4 (-5.0–4.9)	-4.5 (-9.5–1.6)	0.105
Δ SBP [mm Hg]	-3.6 (-6.0–2.4)	-5.5 (-7.7–5.3)	-6.7 (-10.4–1.6)	0.105
Δ DBP [mm Hg]	0.4 (-1.9–1.8)	1.1 (-0.8–4.6)	1.6 (-2.5–3.0)	0.558
Δ SVV [%]	-0.1 (-1.0–0.3)	0.3 (0.2–3.8)^*^	6.4 (4.3–9.6)^*#^	** < 0.001**
Δ CO [L/min]	0.12 (-0.25–0.38)	0.12 (-0.25–0.38)	0.09 (-0.29–0.29)^*#^	** < 0.001**
Δ CVP [mmHg]	0.6 (0.2–1.2)	1.8 (0.5–2.6)^*^	3.2 (2.1–3.7)^*#^	** < 0.001**
Δ PAP [mmHg]	1.9 (0.7–2.2)	4.1 (1.8–6.1)^*^	9.2 (6.0–11.4)^*#^	** < 0.001**
*Autonomic nervous system metrics*				
Δ meanNN [ms]	-21.0 (-33.8– -4.6)	-53.6 (-107.0– -26.7)^*^	-143.4 (-202.8– -84.5)^*#^	** < 0.001**
Δ SDNN [ms]	-2.8 (-3.2–0.5)	-2.9 (-3.9– -0.8)	-0.2 (-5.1–1.3)	0.778
Δ RMSSD [ms]	-2.7 (-3.4–0.4)	-3.1 (-3.8– -0.7)	0.0 (-5.2–1.5)	0.778
Δ GI [%]	0.1 (0.0–0.1)	-0.1 (-0.2–0.1)	0.1 (-0.2–0.0)	0.205
Δ PI [%]	0.2 (-2.0–1.1)	2.3 (-3.1–3.7)	1.2 (-1.7–8.0)	0.338
Δ FuzzyEn [a.u.]	0.1 (-0.1–0.4)	0.0 (-0.2–0.3)	0.0 (-0.2–0.1)	0.097
Δ SampEn [a.u.]	0.3 (-0.1–0.5)	0.2 (-0.2–0.6)	0.0 (-0.2–0.1)	0.173
Δ BRS [ms/mm Hg]	-1.1 (-2.2– -0.4)	-2.4 (-3.5–-1.8)	-2.8 (-4.5– -0.8)	0.097

ICP, intracranial pressure; PRx, pressure reactivity index; EtCO_2_, end tidal carbon dioxide; Paw_ei_, end-inspiratory airway pressure; Paw_ee_, end-expiratory airway pressure; Pes_ei_, end-inspiratory esophageal pressure; Pes_ee_, end-expiratory esophageal pressure; TPP_ei_, end-inspiratory transpulmonary pressure; TPP_ee_, end-expiratory transpulmonary pressure; Ppeak_cw_, peak inspiratory pressure of the chest wall; Ppeak_l_, lung peak pressure; Ppeak_rs_, peak inspiratory pressure of the respiratory system; HR, heart rate; MAP, mean arterial pressure; SBP, systolic arterial pressure; DBP, diastolic arterial pressure; SVV, stroke volume variation; CO, cardiac ouput; CVP, central venous pressure; PAP, pulmonary artery pressure; meanNN, mean intervals between normal R peaks; SDNN, standard deviation of the normal pulse heartbeats; RMSSD, the root mean square of successive differences between normal heartbeats; GI, Guzik’s Index; PI, Porta’s Index; FuzzyEn, fuzzy entropy; SampEn, sample entropy; BRS, baroreflex sensitivity; p-values were determined using Friedman’s ANOVA; post hoc comparisons between three PEEP values (10, 15, and 20 cmH₂O) were performed using Wilcoxon test with a Bonferroni adjustment: * refers to 10 cmH₂O, # refers to 15 cmH₂O; significant results marked in bold

**Table 4:** Changes in parameters at incremental positive end-expiratory pressure PEEP levels (10, 15, and 20 cmH₂O), expressed as differences (Δ) from the baseline level (5 cmH₂O) in the supine position (*n* = 12). Data are presented as the median (upper–lower quartile)

Supine position				
Relative changes of parameter	10 [cmH₂O] *n* = 12	15 [cmH₂O] *n* = 12	20 [cmH₂O] *n* = 12	p
*Neuromonitoring parameters*				
Δ ICP [mm Hg]	1.1 (0.8–1.2)	2.6 (-2.0–7.7)	3.4 (3.1–4.4)^*^	**0.017**
Δ PRx [a.u.]	0.03 (-0.07–0.12)	-0.06 (-0.29–0.33)	-0.15 (-0.36–0.26)	**0.046**
*Respiratory parameters*				
Δ EtCO_2_ [mm Hg]	0.1 (0.1–0.1)	0.3 (0.1–0.8)	0.7 (0.0–0.9)	0.050
Δ respiratory rate [breaths/min]	0.6 (-0.1–1.3)	1.6 (-0.1–3.4)	7.8 (5.7–8.6)^*#^	** < 0.001**
Δ Paw_ei_ [cmH₂O]	6.4 (6.0–6.7)	12.7 (12.2–13.2)	19.3 (18.1–20.5)^*#^	** < 0.001**
Δ Paw_ee_ [cmH₂O]	5.3 (5.2–5.4)	10.5 (10.4–10.7)^*^	15.8 (15.6–16.1)^*#^	** < 0.001**
Δ Pes_ei_ [cmH₂O]	1.4 (0.7–1.8)	3.5 (1.4–4.0)	5.2 (2.7–6.0)^*#^	** < 0.001**
Δ Pes_ee_ [cmH₂O]	2.0 (0.7–2.2)	3.8 (2.1–4.3)^*^	5.4 (3.4–6.7)^*#^	** < 0.001**
Δ TPP_ei_ [cmH₂O]	4.9 (4.7–5.8)	8.9 (8.6–10.3)	14.5 (12.2–16.6)^*#^	** < 0.001**
Δ TPP_ee_ [cmH₂O]	3.5 (3.1–4.5)	6.5 (6.1–8.5)^*^	10.0 (9.2–12.6)^*#^	** < 0.001**
Δ Ppeak_rs_ [cmH₂O]	6.0 (5.6–6.8)	12.3 (12.0–13.5)^*^	20.3 (19.3–22.3)^*#^	** < 0.001**
Δ Ppeak_cw_ [cmH₂O]	1.7 (0.9–1.9)	3.7 (1.8–4.2)	5.6 (3.4–6.5)^*#^	** < 0.001**
Δ Ppeak_l_ [cmH₂O]	4.8 (4.3–5.2)	8.7 (8.5–10.0)^*^	15.0 (13.1–17.3)^*#^	** < 0.001**
*Systemic hemodynamics parameters*				
ΔHR [bpm]	1.9 (0.1–4.7)	14.2 (2.8–32.3)^*^	28.2 (20.2–40.8)^*^	** < 0.001**
Δ MAP [mm Hg]	-0.8 (-4.2–3.9)	2.4 (-0.9–7.6)	1.4 (-1.1–5.8)	0.920
Δ SBP [mm Hg]	-1.4 (-4.6–5.7)	4.4 (-1.6–10.9)	0.1 (-2.6–7.6)	0.174
Δ DBP [mm Hg]	0.2 (-3.2–5.2)	5.2 (1.4–9.2)	5.9 (0.7–10.0)	**0.013**
Δ SVV [%]	1.1 (0.2–1.6)	3.0 (0.3–4.5)^*^	9.0 (4.0–10.5)^*#^	** < 0.001**
Δ CO [L/min]	-0.10 (-0.40–0.20)	-0.30 (-0.81–0.65)	-0.10 (-0.40–0.20)	0.322
Δ CVP [mmHg]	1.0 (0.6–1.3)	2.5 (2.1–3.2)^*^	3.4 (2.7–4.5)^*^	** < 0.001**
Δ PAP [mmHg]	2.4 (1.1–2.9)	6.0 (3.8–8.1)^*^	9.6 (7.4–11.7)^*#^	** < 0.001**
*Autonomic nervous system metrics*				
Δ meanNN [ms]	-17.4 (-42.3– -0.9)	-90.56 (-230.5– -21.5)^*^	-149.0 (-254.2– -109.5)^*#^	**0.001**
Δ SDNN [ms]	-1.8 (-4.0– -0.8)	-2.9 (-11.5– -0.1)	-2.9 (-13.4–3.8)	0.367
Δ RMSSD [ms]	-1.7 (-3.9– -0.8)	-2.9 (-11.2– 0.1)	-2.7 (-12.8–4.0)	0.367
Δ GI [%]	-0.1 (-0.2–0.0)	-0.2 (-0.3–0.0)	-0.2 (-0.5–0.0)	0.075
Δ PI [%]	-0.8 (-3.4–1.0)	1.8 (-2.5–6.5)	2.0 (-0.6–4.5)	0.105
Δ FuzzyEn [a.u.]	0.2 (-0.2– 0.3)	0.1 (-0.1– 0.4)	-0.3 (-0.5– -0.1)^*#^	**0.006**
Δ SampEn [a.u.]	0.2 (-0.4– 0.4)	0.3 (-0.1–0.8)	-0.4 (-0.1–0.8)^*#^	**0.018**
Δ BRS [ms/mm Hg]	-1.6 (-4.9– -0.3)	-1.9 (-8.2– -1.3)	-3.9 (-7.6– -0.2)^*^	**0.013**

ICP, intracranial pressure; PRx, pressure reactivity index; EtCO_2_, end tidal carbon dioxide; Paw_ei_, end-inspiratory airway pressure; Paw_ee_, end-expiratory airway pressure; Pes_ei_, end-inspiratory esophageal pressure; Pes_ee_, end-expiratory esophageal pressure; TPP_ei_, end-inspiratory transpulmonary pressure; TPP_ee_, end-expiratory transpulmonary pressure; Ppeak_cw_, peak inspiratory pressure of the chest wall; Ppeak_l_, lung peak pressure; Ppeak_rs_, peak inspiratory pressure of the respiratory system; HR, heart rate; MAP, mean arterial pressure; SBP, systolic arterial pressure; DBP, diastolic arterial pressure; SVV, stroke volume variation; CO, cardiac output; CVP, central venous pressure; PAP, pulmonary artery pressure; meanNN, mean intervals between normal R peaks; SDNN, standard deviation of the normal pulse heartbeats; RMSSD, the root mean square of successive differences between normal heartbeats; GI, Guzik’s Index; PI, Porta’s Index; FuzzyEn, fuzzy entropy; SampEn, sample entropy; BRS, baroreflex sensitivity; p values were determined using Friedman’s ANOVA; post hoc comparisons between three PEEP values (10, 15, and 20 cmH₂O) were performed using Wilcoxon test with a Bonferroni adjustment: * refers to 10 cmH₂O, # refers to 15 cmH₂O; significant results marked in bold

### Effects of PEEP modulation on ANS activity

**Fig. 2:**
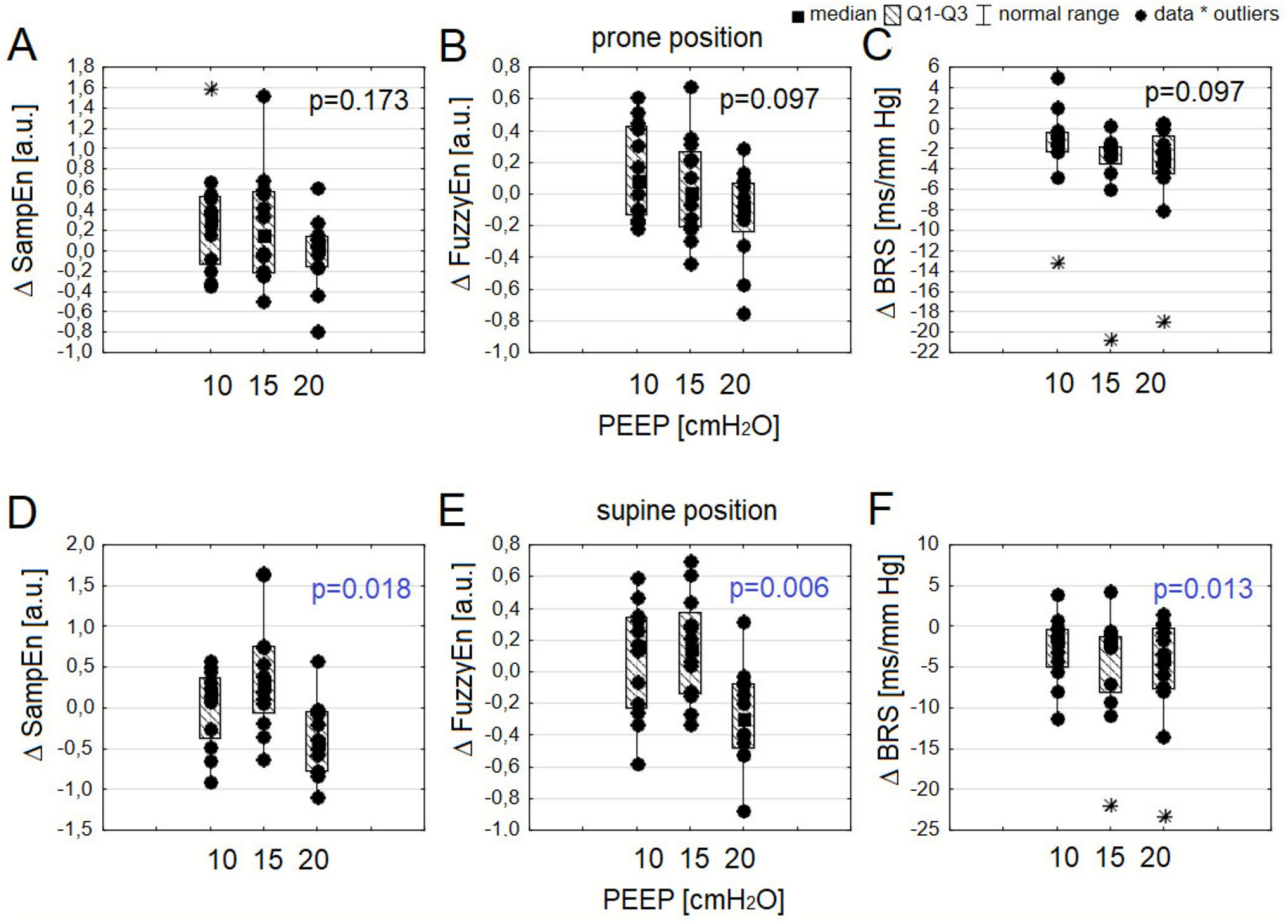
Boxplots showing changes in sample entropy (SampEn), fuzzy entropy (FuzzyEn), and baroreflex sensitivity (BRS) at incremental positive end-expiratory pressure (PEEP) levels (10, 15, and 20 cmH₂O), expressed as differences (Δ) from baseline (5 cmH₂O) in the prone position (**A-C**) and supine position (**D-F**)

The effects of incremental PEEP levels on HRV and BRS parameters are presented for the prone (Table 3) and supine (Table 4) positions. In the prone position, meanNN decreased significantly with increasing PEEP (p < 0.001), reflecting a progressive increase in HR. Time-domain HRV metrics (SDNN and RMSSD), as well as heart rhythm asymmetry indices (GI and PI), did not change significantly across PEEP levels. The entropy metrics SampEn and FuzzyEn (Fig. 2 A-B), as well as BRS (Fig. 2 C), tended to decrease with increasing PEEP. In the supine position, mean NN decreased significantly with increasing PEEP (p < 0.001). Time-domain HRV metrics (SDNN and RMSSD), as well as heart rhythm asymmetry indices (GI and PI), did not change significantly across PEEP levels. The entropy metrics SampEn (p = 0.018, Fig. 2 D) and FuzzyEn (p = 0.006, Fig. 2 E) decreased with increasing PEEP, and post hoc tests indicated that the reduction at 20 cmH₂O was greater than that at 10 cmH₂O (SampEn: p = 0.012 and FuzzyEn: p = 0.005) and 15 cmH₂O (SampEn: p = 0.005 and FuzzyEn: p = 0.006). BRS also decreased with increasing PEEP (p = 0.013), with post hoc analysis showing that the decrease at 20 cmH₂O was substantially greater than that at 10 cmH₂O (p = 0.012) (Fig. 2F). The relative differences in ANS metrics at each PEEP level (10, 15, and 20 cmH₂O) between the prone and supine positions are presented in the Supplementary Materials (Supplementary Table 1). In general, there were no relative differences in ANS parameters caused by the change from prone to supine position, except for ΔFuzzyEn and ΔSampEn at PEEP of 20 cmH₂O.

### Associations between ANS activity and cerebrovascular reactivity

Univariate LMEM was applied to assess the association between cerebrovascular reactivity and ANS activity (Table 5). In both the prone and supine positions, all the analyzed ANS metrics significantly influenced PRx. The multivariate LMEM, adjusted for PEEP level, was applied to assess the association between cerebrovascular reactivity and ANS activity (Table 6). In the prone position, SDNN and RMSSD were highly predictive of PRx (p = 0.001 for both). Heart rhythm asymmetry metrics (GI and PI) were also significantly associated with the PRx (p = 0.003 and p = 0.007, respectively). HRV entropy metrics (FuzzyEn and SampEn) and BRS did not significantly influence PRx after adjustment for PEEP. In the supine position, the ANS parameters had no significant effect on PRx after adjustment for PEEP (Table 6).

**Table 5:** Univariate linear mixed effects models (LMEMs) explaining the variation in the pressure reactivity index (PRx) by autonomic nervous system metrics using pooled data. Significant factors are indicated in bold

Body position	Response variable	Predictor	F	p
Prone	PRx	HR [bpm]	27.52	** < 0.001**
Prone	PRx	SBP [mmHg]	27.52	** < 0.001**
Prone	PRx	DBP [mmHg]	29.34	** < 0.001**
Prone	PRx	meanNN [ms]	23.89	** < 0.001**
Prone	PRx	SDNN [ms]	4.24	**0.045**
Prone	PRx	RMSSD [ms]	4.45	**0.041**
Prone	PRx	GI [%]	30.67	** < 0.001**
Prone	PRx	PI [%]	28.90	** < 0.001**
Prone	PRx	FuzzyEn [a.u.]	6.76	**0.013**
Prone	PRx	SampEn [a.u.]	7.72	**0.008**
Prone	PRx	BRS [ms/mmHg]	6.53	**0.014**
Supine	PRx	HR [bpm]	43.45	** < 0.001**
Supine	PRx	SBP [mmHg]	30.46	** < 0.001**
Supine	PRx	DBP [mmHg]	41.49	** < 0.001**
Supine	PRx	meanNN [ms]	27.83	** < 0.001**
Supine	PRx	SDNN [ms]	22.80	** < 0.001**
Supine	PRx	RMSSD [ms]	23.13	** < 0.001**
Supine	PRx	GI [%]	37.84	** < 0.001**
Supine	PRx	PI [%]	37.64	** < 0.001**
Supine	PRx	FuzzyEn [a.u.]	23.58	**0.001**
Supine	PRx	SampEn [a.u.]	14.28	**0.001**
Supine	PRx	BRS [ms/mmHg]	11.82	**0.001**

PRx, pressure reactivity index; HR, heart rate; SBP, systolic blood pressure; DBP, diastolic blood pressure; meanNN, intervals between normal R peaks; SDNN, standard deviation of the normal pulse heartbeats; RMSSD, root mean square of successive differences between normal heartbeats; GI, Guzik’s Index; PI, Porta’s Index; FuzzyEn, fuzzy entropy; SampEn, sample entropy; BRS, baroreflex sensitivity

**Table 6:** Multivariate linear mixed effects models (LMEMs) explaining the variation in the pressure reactivity index (PRx) by autonomic nervous system metrics, adjusted by positive end-expiratory pressure (PEEP) levels using pooled data. Significant factors are indicated in bold

Body position	Response variable	Predictor 1	F	p	Predictor 2	F	p
Prone	PRx	HR [bpm]	16.43	** < 0.001**	PEEP [cmH₂O]	4.84	**0.035**
Prone	PRx	SBP [mmHg]	8.31	**0.008**	PEEP [cmH₂O]	0.78	0.384
Prone	PRx	DBP [mmHg]	10.98	**0.003**	PEEP [cmH₂O]	1.75	0.196
Prone	PRx	meanNN [ms]	3.90	0.057	PEEP [cmH₂O]	0.17	0.682
Prone	PRx	SDNN [ms]	13.21	**0.001**	PEEP [cmH₂O]	34.93	** < 0.001**
Prone	PRx	RMSSD [ms]	12.03	**0.001**	PEEP [cmH₂O]	32.88	** < 0.001**
Prone	PRx	GI [%]	10.70	**0.003**	PEEP [cmH₂O]	2.04	0.166
Prone	PRx	PI [%]	8.65	**0.007**	PEEP [cmH₂O]	1.53	0.226
Prone	PRx	FuzzyEn [a.u.]	2.36	0.133	PEEP [cmH₂O]	15.04	**0.001**
Prone	PRx	SampEn [a.u.]	0.62	0.435	PEEP [cmH₂O]	11.18	**0.003**
Prone	PRx	BRS [ms/mmHg]	0.10	0.765	PEEP [cmH₂O]	11.52	**0.002**
Supine	PRx	HR [bpm]	2.58	0.116	PEEP [cmH₂O]	0.01	0.906
Supine	PRx	SBP [mmHg]	1.19	0.281	PEEP [cmH₂O]	4.51	**0.046**
Supine	PRx	DBP [mmHg]	2.91	0.098	PEEP [cmH₂O]	0.24	0.628
Supine	PRx	meanNN [ms]	0.17	0.687	PEEP [cmH₂O]	5.21	**0.030**
Supine	PRx	SDNN [ms]	0.15	0.702	PEEP [cmH₂O]	10.71	**0.002**
Supine	PRx	RMSSD [ms]	0.17	0.689	PEEP [cmH₂O]	10.54	**0.003**
Supine	PRx	GI [%]	1.19	0.285	PEEP [cmH₂O]	0.50	0.484
Supine	PRx	PI [%]	1.40	0.246	PEEP [cmH₂O]	0.35	0.558
Supine	PRx	FuzzyEn [a.u.]	0.90	0.349	PEEP [cmH₂O]	7.18	**0.016**
Supine	PRx	SampEn [a.u.]	0.01	0.936	PEEP [cmH₂O]	14.87	**0.001**
Supine	PRx	BRS [ms/mmHg]	0.01	0.943	PEEP [cmH₂O]	20.34	** < 0.001**

PRx, pressure reactivity index; HR, heart rate; SBP, systolic blood pressure; DBP, diastolic blood pressure; meanNN, intervals between normal R peaks; SDNN, standard deviation of the normal pulse heartbeats; RMSSD, root mean square of successive differences between normal heartbeats; GI, Guzik’s Index; PI, Porta’s Index; FuzzyEn, fuzzy entropy; SampEn, sample entropy; BRS, baroreflex sensitivity

## Discussion

This study presents the effects of increased intrathoracic pressures, induced by means of incremental PEEP increases on ANS activity and investigates its relationship with cerebrovascular reactivity in an experimental model. Mean arterial blood pressure, systolic blood pressure, and cardiac output stayed stable as PEEP increased, but heart rate rose (shorter meanNN), suggesting compensated autonomic drive. Blood pressure alone may therefore underestimate the cardiovascular stress of higher PEEP, so HRV trends should be followed as an added tolerance signal. PEEP also significantly affected HRV entropy metrics and BRS in the supine position, with similar decreasing trends observed in the prone position. In the univariate LMEM analyses, all the ANS metrics significantly influenced PRx in both positions. However, in multivariate LMEM adjusted for PEEP, only time-domain HRV metrics and heart rate asymmetry indices remained significantly associated with PRx in the prone position, whereas no ANS metrics remained significant in the supine position.

A PEEP-associated fall in BRS indicates a reduced capacity to buffer short-term blood pressure fluctuations, even if average mean arterial pressure stays unchanged. Blood pressure fluctuations may matter for organ perfusion when autoregulatory reserve is limited [51]. Increasing PEEP has been shown to decrease baroreflex sensitivity in mechanically ventilated patients, aligning with our findings [79]. A steady pattern of interbeats interval variability, in both amplitude and phase, is considered indicative of a blunted autonomic response [79]. Valipour et al. demonstrated a significant reduction in the mean slope of spontaneous baroreceptor sequences at nasal positive airway pressure (nPAP) levels above 10 cmH₂O compared with lower levels (0, 3, or 5 cmH₂O). This finding suggests that the ability of the arterial baroreflex to modulate pulse interbeat interval responses to changes in systolic blood pressure diminishes as vagal activity is withdrawn under high nPAP levels [77]. In healthy awake subjects, Fietze et al. reported that PEEP at 5 cmH₂O increases blood pressure, decreases HR, and enhances the high-frequency component of the α-index of baroreflex gain, reflecting enhanced parasympathetic regulation of heart rate in response to blood pressure changes [26]. Garet et al. reported that the sympathetic spontaneous cardiac baroreflex drive, represented by the low-frequency components of the α-index of baroreflex gain, appears to transitorily decrease under inspiratory pressure support plus PEEP ventilation [27]. The authors suggested that this inhibition of the sympathetic control of heart rate, evidenced by decreases in low-frequency baroreflex gain, may result from stimulation of pulmonary stretch receptors due to assisted ventilation-induced increases in end-expiratory lung volume [27]. In our study, we observed a diminished cross-correlation BRS at higher PEEP levels. A decrease in baroreflex activity may indicate more challenging blood pressure regulation, potentially affecting cerebral perfusion.

HRV and reduced entropy have also been associated with outcome in trauma patients and critical illness cohorts [7, 8, 48, 50]. Decreased entropy and complexity indices of ICP and HR have been associated with unfavorable outcomes or mortality in various diseases, including traumatic brain injury [43, 87]. The complex dynamic behavior and nonlinear characteristics of the cardiovascular control system necessitate the use of advanced nonlinear and nonstationary analytical methods to capture information hidden within pulse heartbeat interval time series. [84]. HRV entropy indices provide valuable insights beyond traditional time-domain and frequency-domain HRV metrics by evaluating the complexity or irregularity of the time series [42]. As healthy heart rhythms are inherently complex and unpredictable, low HRV entropy indicates that the heart’s response is less complex, more rigid, and less able to adapt to changing demands—a pattern observed in critically ill patients [18, 60, 85]. In our study, we observed a decrease in HRV entropy with increasing PEEP, with the most pronounced changes observed between 10 cmH₂O and 20 cmH₂O. Reduced HRV entropy (SampEn and FuzzyEn) indicates a loss of complexity/irregularity in the pulse interval time series, consistent with reduced autonomic flexibility and diminished capacity to adapt to changing physiologic demands. In this context, falling HRV entropy with increasing PEEP may represent an early signal of reduced physiologic reserve—potentially detectable even when mean arterial pressure and systolic blood pressure remain stable.

In our study, ANS metrics were associated with PRx in univariate models using pooled data. However, after adjustment by PEEP, only selected ANS metrics, specifically, the time-domain parameters (SDNN and RMSSD) and heart rate asymmetry indices (GI and PI), remained significant. Previous studies have shown that heart rate asymmetry captures patterns in heart rate dynamics beyond the respiratory cycle, potentially reflecting more complex autonomic interactions and distinguishing different autonomic responses to physiological changes [24]. The lack of independent significance for other ANS measures may reflect the limited statistical power of the proposed model to detect modest autonomic contributions against the stronger effect of PEEP. Alternatively, if PEEP influences both ANS activity and intracranial hemodynamics, the observed associations of the ANS with PRx may be mediated through PEEP-dependent pathways.

The cardiopulmonary effects of prone positioning, including improved lung mechanics and a more homogenous ventilation-perfusion distribution, are well established [54]. In our study, prone positioning was associated with preserved ANS metrics and preserved autonomic modulation of cerebrovascular reactivity during incremental PEEP. Notably, heart rate was slightly higher at baseline in the prone position, suggesting reduced preload and a relative predominance of sympathetic activity, while cardiac output and arterial blood pressure were comparable between positions. One potential explanation for the preserved autonomic modulation under PEEP is that this relative sympathetic activation may help maintain cerebrovascular responsiveness during PEEP-induced increases in intrathoracic pressure. In addition, because of gravitational effects, chest wall pressure is lower in the prone position, resulting in higher transpulmonary pressure for a given airway pressure. Higher transpulmonary pressure may promote more homogenous lung recruitment and ventilation-perfusion matching, which could stabilize chemical changes and modulate pulmonary mechanoreceptor afferent input to the brainstem. This could enhance heart–lung interaction and reflex signaling to the brain, potentially contributing to the preservation of cerebrovascular reactivity. To our knowledge, this is the first study describing the interaction between ANS and PRx in the prone position; therefore, any mechanistic interpretation remains speculative, as the present study was not designed to study the underlying mechanisms.

Increased intrathoracic pressure can reduce venous return, particularly in hypovolemic states, in which heart rate typically increases. However, if this compensatory mechanism is insufficient, cardiac output and blood pressure may decline. In our study, heart rate increased while cardiac output and blood pressure were maintained. Therefore, our findings should be interpreted in the context of a predominantly euvolemic state. Nonetheless, the reduction in venous return, which likely occurs to some extent with increased intrathoracic pressure, may also influence ANS activity.

Although an established relationship exists between heart rate and HRV [46], numerous studies have demonstrated that HRV has independent prognostic value in patients with cardiovascular disease and provides insight into cardiac autonomic regulation beyond that obtainable from mean heart rate alone [71, 80]. Importantly, certain measures, such as entropy-based metrics used in this study, are less dependent on the alterations in mean heart rate [14, 15].

Taken together, rising heart rate with reduced baroreflex sensitivity and entropy suggests that higher PEEP can potentially increase vulnerability to routine ICU stressors (position changes, suctioning, recruitment maneuvers, or sedative/vasopressor adjustments) even when blood pressure appears unchanged. The presence of similar directional trends in the prone position implies that these autonomic effects may persist across posture, supporting the use of within-patient autonomic trends as complementary bedside signals when individualizing PEEP and judging tolerance.

Some limitations should be acknowledged. This study was conducted in an experimental animal model, which may limit the generalizability of the observations to human patients. However, the swine model is commonly used in cardiovascular research, as the ratio of heart weight to body weight in 20–30 kg pigs is similar to that in adult humans [41]. Moreover, swine closely resemble humans in terms of hemodynamic parameters [41]. Anatomical features, particularly the high position of the moderator band, may explain the faster activation of the right ventricle in the pig heart [19] and the greater sinus rhythm (HR) in pigs than in humans [6, 41]. Nevertheless, the suitability and effectiveness of the swine model for neurotrauma research have been well demonstrated [64] [36] [89]. Autonomic metrics may be influenced by sedation, anesthetic agents, and ventilatory mode. However, as anesthetic agents, dosing, and depth of sedation were kept constant throughout baseline and across PEEP levels, any such effect would represent a consistent bias across all measurements. The number of observations was limited, which reduced the statistical power and may have affected the robustness of the analyses. HRV metrics were estimated from pulse heartbeat intervals derived from ABP, as reliable ECG recordings were not available at certain PEEP levels in four of the twelve cases. Frequency-domain metrics were not included because the appropriate spectral ranges for swine are not standardized [70, 90].

## Conclusions

Changes in intrathoracic pressure, caused by incremental PEEP levels, impact on autonomic activity, as indicated by reduced baroreflex sensitivity and entropy of heart rate variability. Autonomic nervous system metrics could complement blood pressure when evaluating hemodynamic reserve during PEEP titration.

## Supplementary Information

Below is the link to the electronic supplementary material.

Supplementary file1 (DOCX 26 KB)

## Data Availability

The raw data supporting the conclusions of this article will be made available by the authors without undue reservation.
