# Supplementary material for: Impact of positive end-expiratory pressure on autonomic nervous system activity and its interaction with cerebrovascular reactivity – an experimental study
Source: J Clin Monit Comput. 2026 Feb 18;40(3):703–20. doi: 10.1007/s10877-026-01420-4 (PMC13194303; doi:10.1007/s10877-026-01420-4)
Supplement: Supplementary file 1 — Supplementary file1 (DOCX 26 KB) [file 10877_2026_1420_MOESM1_ESM.docx]

**Supplementary Materials**

**Impact of positive end-expiratory pressure on autonomic nervous system activity and its interaction with cerebrovascular reactivity – an experimental study**

**Agnieszka Uryga^1^, Magdalena Kasprowicz^1^, Marek Czosnyka^2^, Agnieszka Kazimierska^1^, Rønnaug Hammervold^3,4,5^, Shirin K. Frisvold^4,6*^**

1. Department of Biomedical Engineering, Faculty of Fundamental Problems of Technology, Wroclaw University of Science and Technology, Wroclaw, Poland

2. Division of Neurosurgery, Department of Clinical Neurosciences, University of Cambridge, Cambridge, United Kingdom

3. Department of Anesthesia and Intensive Care, Nordland Hospital Trust, Bodø, Norway

4. Department of Clinical Medicine, Faculty of Health Sciences, UiT The Arctic University of Norway, Tromsø, Norway

5. Research Laboratory, Nordland Hospital Trust, Bodø, Norway

6. Department of Anesthesia and Intensive Care, University Hospital North Norway, Tromso, Norway

*corresponding author

Contact details:

Shirin K. Frisvold

e-mail: shirin.frisvold@uit.no

Department of Clinical Medicine, Faculty of Health Sciences, UiT The Arctic University of Norway, Tromsø, Norway

**Effects of PEEP modulation on neuromonitoring parameters**

Changes in neuromonitoring parameters across incremental PEEP levels are presented for the prone (Table 3, main manuscript) and supine (Table 4, main manuscript) positions in all pigs (*n* = 12). In the prone position, ICP increased significantly with increasing PEEP (p<0.001), and post hoc tests indicated that the change at 20 cmH₂O was substantially greater than that at 10 cmH₂O (p = 0.008) and 15 cmH₂O (p = 0.012) without affecting cerebral autoregulation. PEEP level had an overall effect on EtCO_2_ (p = 0.013). However, no significant pairwise differences were detected in post hoc comparisons. Changes in the respiratory rate significantly increased with increasing PEEP (p = 0.004). HR also increased with increasing PEEP (p < 0.001), and the change at 20 cmH₂O was significantly greater than that at 10 cmH₂O (p = 0.004) and 15 cmH₂O (p = 0.002), with no effect on MAP, SBP, or DBP. Significant changes were also observed in SVV (p < 0.001).

In the supine position, ICP increased significantly with increasing PEEP (p = 0.017), and the change at 20 cmH₂O was greater than that at 10 cmH₂O (p = 0.003). The effect of PEEP on cerebrovascular reactivity was borderline significant (p = 0.046), with more negative PRx values (indicating better cerebrovascular reactivity), although no significant pairwise differences were detected in post hoc comparisons. The respiratory rate increased significantly with increasing PEEP (p < 0.001), with no accompanying effect on EtCO_2_. HR increased with increasing PEEP (p < 0.001), and post hoc analysis revealed that the change at 20 cmH₂O was greater than that at 10 cmH₂O (p = 0.002). MAP and SBP remained unchanged, whereas DBP was significantly elevated (p = 0.013) and SVV increased with increasing PEEP (p < 0.001).

The relative differences in neuromonitoring parameters at each PEEP level (10, 15, and 20 cmH₂O) between the prone and supine positions are presented in the Supplementary Table 1. Body position change from prone to supine did not cause relative changes in neuromonitoring parameters, except for SVV at PEEP of 10 cm H₂O.

**Supplementary Table 1.** Relative differences in neuromonitoring parameters at each positive end-expiratory pressure (PEEP) level (10, 15, and 20 cmH₂O) between the prone and supine positions within the same pig. Comparisons were performed using the Wilcoxon test. Data are presented as the median (upper–lower quartile).

|  | **Prone position**  **PEEP**  **(*n* = 12)** | | | **Supine position**  **PEEP**  **(*n* = 12)** | | | **Wilcoxon p-value**  **(PEEP value)** | | |
| --- | --- | --- | --- | --- | --- | --- | --- | --- | --- |
| **Parameter** | **10 [cmH_2_O]** | **15 [cmH_2_O]** | **20 [cmH_2_O]** | **10 [cmH_2_O]** | **15 [cmH_2_O]** | **20 [cmH_2_O]** | **p (10)** | **p (15)** | **p (20)** |
| *Neuromonitoring parameters* | | | | | | | | | |
| **Δ ICP**  **[mm Hg]** | 0.5  (0.2–1.5) | 1.4  (0.6–2.5) | 2.3  (1.4–4.1) | 1.1  (0.8–1.2) | 2.6  (-2.0–7.7) | 3.4  (3.1–4.4) | 0.388 | 0.694 | 0.182 |
| **Δ PRx**  **[a.u.]** | 0.15  (0.11–0.20) | 0.18  (0.13–0.32) | 0.19  (0.0–0.30) | 0.03  (-0.07–0.12) | -0.06  (-0.29–0.33) | -0.15  (-0.36–0.26) | 0.071 | 0.388 | 0.272 |
| *Respiratory parameters* | | | | | | | | | |
| **Δ EtCO_2_**  **[mm Hg]** | 0.1  (-0.1–0.1) | 0.1  (0.0–0.5) | 0.4  (0.1–0.6) | 0.1  (0.1–0.1) | 0.3  (0.1–0.8) | 0.7  (0.0–0.9) | 0.638 | 0.694 | 0.638 |
| **Δ RR**  **[breaths/min]** | 0.4  (-0.2–2.0) | 2.2  (-0.2–4.6) | 9.9  (2.3–14.6) | 0.6  (-0.1–1.3) | 1.6  (-0.1–3.4) | 7.8  (5.7–8.6) | 0.583 | 0.388 | 0.388 |
| **Δ Paw_ei_**  **[cmH₂O]** | 5.3  (4.7–6.1) | 11.7  (10.9–12.9) | 19.9  (18.2–22.0) | 6.4  (6.0–6.7) | 12.7  (12.2–13.2) | 19.3  (18.1–20.5) | **0.034** | 0.346 | 0.530 |
| **Δ Paw_ee_**  **[cmH₂O]** | 5.5  (5.3–5.7) | 10.8  (10.5–11.0) | 16.0  (15.7–16.2) | 5.3  (5.2–5.4) | 10.5  (10.4–10.7) | 15.8  (15.6–16.1) | 0.136 | **0.015** | 0.937 |
| **Δ Pes_ei_**  **[cmH₂O]** | 1.0  (-0.5–1.7) | 4.2  (2.7–5.0) | 5.2  (3.3–7.7) | 1.4  (0.7–1.8) | 3.5  (1.4–4.0) | 5.2  (2.7–6.0) | 0.136 | 0.347 | 0.814 |
| **Δ Pes_ee_**  **[cmH₂O]** | 1.6  (0.7–2.8) | 4.5  (2.7–6.2) | 5.6  (3.4–8.1) | 2.0  (0.7–2.2) | 3.8  (2.1–4.3) | 5.4  (3.4–6.7) | 0.875 | 0.272 | 0.875 |
| **Δ TPP_ei_**  **[cmH₂O]** | 4.4  (3.0–5.2) | 8.4  (6.2–9.8) | 13.7  (10.6–17.6) | 4.9  (4.7–5.8) | 8.9  (8.6–10.3) | 14.5  (12.2–16.6) | 0.209 | 0.308 | 0.937 |
| **Δ TPP_ee_**  **[cmH₂O]** | 3.4  (2.4–4.4) | 6.2  (4.5–8.1) | 9.7  (7.2–11.8) | 3.5  (3.1–4.5) | 6.5  (6.1–8.5) | 10.0  (9.2–12.6) | 0.875 | 0.433 | 0.530 |
| **Δ Ppeak_rs_**  **[cmH₂O]** | 5.4  (4.5–6.2) | 11.1  (10.6–14.7) | 20.9  (18.7–24.1) | 6.0  (5.6–6.8) | 12.3  (12.0–13.5) | 20.3  (19.3–22.3) | **0.028** | 0.875 | 0.530 |
| **Δ Ppeak_cw_**  **[cmH₂O]** | 1.3  (0.6–1.8) | 4.1  (2.9–5.4) | 5.9  (4.0–8.2) | 1.7  (0.9–1.9) | 3.7  (1.8–4.2) | 5.6  (3.4–6.5) | 0.182 | 0.694 | 0.937 |
| **Δ Ppeak_l_**  **[cmH₂O]** | 4.0  (3.7–5.1) | 7.8  (5.8–11.6) | 14.5  (10.6–18.8) | 4.8  (4.3–5.2) | 8.7  (8.5–10.0) | 15.0  (13.1–17.3) | 0.182 | 0.638 | 0.937 |
|  | | | | | | | | | |
|  | **Prone position PEEP**  **(*n* = 12)** | | | **Supine position PEEP**  **(*n* = 12)** | | | **Wilcoxon p-value**  **(PEEP value)** | | |
| **Parameter** | **10 [cmH_2_O]** | **15 [cmH_2_O]** | **20 [cmH_2_O]** | **10 [cmH_2_O]** | **15 [cmH_2_O]** | **20 [cmH_2_O]** | **p (10)** | **p (15)** | **p (20)** |
| *Systemic hemodynamics parameters* | | | | | | | | | |
| **Δ HR**  **[bpm]** | 4.0  (0.3–6.2) | 10.4  (3.6–17.6) | 25.4  (20.0–45.7) | 1.9  (0.1–4.7) | 14.2  (2.8–32.3) | 28.2  (20.2–40.8) | 0.937 | 0.694 | 0.937 |
| **Δ MAP**  **[mm Hg]** | -0.2  (-4.5–3.0) | 0.4  (-5.0–4.9) | -4.5  (-9.5–1.6) | -0.8  (-4.2–3.9) | 2.4  (-0.9–7.6) | 1.4  (-1.1–5.8) | 0.875 | 0.239 | 0.239 |
| **Δ SBP**  **[mm Hg]** | -3.6  (-6.0–2.4) | -5.5  (-7.7–5.3) | -6.7  (-10.4–1.6) | -1.4  (-4.6–5.7) | 4.4  (-1.6–10.9) | 0.1  (-2.6–7.6) | 0.209 | 0.308 | 0.308 |
| **Δ DBP**  **[mm Hg]** | 0.4  (-1.9–1.8) | 1.1  (-0.8–4.6) | 1.6  (-2.5–3.0) | 0.2  (-3.2–5.2) | 5.2  (1.4–9.2) | 5.9  (0.7–10.0) | 0.875 | 0.347 | 0.117 |
| **Δ SVV**  **[%]** | -0.1  (-1.0–0.3) | 0.3  (0.2–3.8) | 6.4  (4.3–9.6) | 1.1  (0.2–1.6) | 3.0  (0.3–4.5) | 9.0  (4.0–10.5) | **0.019** | 0.583 | 0.583 |
| **Δ CO**  **[L/min]** | 0.12  (-0.25–0.38) | 0.12  (-0.25–0.38) | 0.09  (-0.29–0.29) | -0.10  (-0.40–0.20) | -0.30  (-0.81–0.65) | -0.10  (-0.40–0.20) | 0.408 | 0.530 | 0.480 |
| **Δ CVP**  **[mmHg]** | 0.6  (0.2–1.2) | 1.8  (0.5–2.6) | 3.2  (2.1–3.7) | 1.0  (0.6–1.3) | 2.5  (2.1–3.2) | 3.4  (2.7–4.5) | **0.005** | **0.005** | **0.003** |
| **Δ PAP**  **[mmHg]** | 1.9  (0.7–2.2) | 4.1  (1.8–6.1) | 9.2  (6.0–11.4) | 2.4  (1.1–2.9) | 6.0  (3.8–8.1) | 9.6  (7.4–11.7) | 0.157 | 0.100 | 0.347 |
| *Autonomic nervous system metrics* | | | | | | | | | |
| **Δ meanNN**  **[ms]** | -21.0  (-33.8– -4.6) | -53.6  (-107.0– -26.7) | -143.4  (-202.8– -84.5) | -17.4  (-42.3– -0.9) | -90.56  (-230.5– -21.5) | -149.0  (-254.2– -109.5) | 0.694 | 0.638 | 0.307 |
| **Δ SDNN**  **[ms]** | -2.8  (-3.2–0.5) | -2.9  (-3.9– -0.8) | -0.2  (-5.1–1.3) | -1.8  (-4.0– -0.8) | -2.9  (-11.5– -0.1) | -2.9  (-13.4–3.8) | 0.814 | 0.583 | 0.480 |
| **Δ RMSSD**  **[ms]** | -2.7  (-3.4–0.4) | -3.1  (-3.8– -0.7) | 0.0  (-5.2–1.5) | -1.7  (-3.9– -0.8) | -2.9  (-11.2– 0.1) | -2.7  (-12.8–4.0) | 0.814 | 0.638 | 0.480 |
| **Δ GI**  **[%]** | 0.1  (0.0–0.1) | -0.1  (-0.2–0.1) | 0.1  (-0.2–0.0) | -0.1  (-0.2–0.0) | -0.2  (-0.3–0.0) | -0.2  (-0.5–0.0) | 0.158 | 0.158 | 0.272 |
| **Δ PI**  **[%]** | 0.2  (-2.0–1.1) | 2.3  (-3.1–3.7) | 1.2  (-1.7–8.0) | -0.8  (-3.4–1.0) | 1.8  (-2.5–6.5) | 2.0  (-0.6–4.5) | 0.347 | 0.209 | 0.754 |
| **Δ FuzzyEn**  **[a.u.]** | 0.1  (-0.1–0.4) | 0.0  (-0.2–0.3) | 0.0  (-0.2–0.1) | 0.2  (-0.2– 0.3) | 0.1  (-0.1– 0.4) | -0.3  (-0.5– -0.1) | 0.530 | 0.480 | **<0.001** |
| **Δ SampEn**  **[a.u.]** | 0.3  (-0.1–0.5) | 0.2  (-0.2–0.6) | 0.0  (-0.2–0.1) | 0.2  (-0.4– 0.4) | 0.3  (-0.1–0.8) | -0.4  (-0.1–0.8) | 0.182 | 0.389 | **0.005** |
| **Δ BRS**  **[ms/mm Hg]** | -1.1  (-2.2– -0.4) | -2.4  (-3.5–-1.8) | -2.8  (-4.5– -0.8) | -1.6  (-4.9– -0.3) | -1.9  (-8.2– -1.3) | -3.9  (-7.6– -0.2) | 0.875 | 0.875 | 0.695 |

*Abbreviations:* ICP, intracranial pressure; PRx, pressure reactivity index; EtCO_2_, end tidal carbon dioxide; Paw_ei_, end-inspiratory airway pressure; Paw_ee_, end-expiratory airway pressure; Pes_ei_, end-inspiratory esophageal pressure; Pes_ee_, end-expiratory esophageal pressure; TPP_ei_, end-inspiratory transpulmonary pressure; TPP_ee_, end-expiratory transpulmonary pressure; Ppeak_cw_, peak inspiratory pressure of the chest wall; Ppeak_l_, lung peak pressure; Ppeak_rs_, peak inspiratory pressure of the respiratory system; HR, heart rate; MAP, mean arterial pressure; SBP, systolic arterial pressure; DBP, diastolic arterial pressure; SVV, stroke volume variation; CO, cardiac output; CVP, central venous pressure; PAP, pulmonary artery pressure; meanNN, mean intervals between normal R peaks; SDNN, the standard deviation of the RR of normal sinus beats; RMSSD, the root mean square of successive differences between normal heartbeats; GI, Guzik’s Index; PI, Porta’s Index; FuzzyEn, fuzzy entropy; SampEn, sample entropy; BRS, baroreflex sensitivity; p values were determined using Wilcoxon test.
